# Born too soon in a resource-limited setting: A 10-year mixed methods review of a special care baby unit for refugees and migrants on the Myanmar-Thailand border

**DOI:** 10.3389/fpubh.2023.1144642

**Published:** 2023-04-12

**Authors:** Ahmar Hashmi, Mu Chae Darakamon, Ko Ko Aung, Mu Mu, Prapatsorn Misa, Podjanee Jittamala, Cindy Chu, Aung Pyae Phyo, Claudia Turner, Francois Nosten, Rose McGready, Verena I. Carrara

**Affiliations:** ^1^Institute for Implementation Science, University of Texas Health Sciences Center (UTHealth), Houston, TX, United States; ^2^Department of Health Promotion and Behavioral Sciences, School of Public Health, University of Texas Health Sciences Center (UTHealth), Houston, TX, United States; ^3^Shoklo Malaria Research Unit, Mahidol-Oxford Tropical Medicine Research Unit, Faculty of Tropical Medicine, Mahidol University, Mae Sot, Thailand; ^4^Faculty of Tropical Medicine, Mahidol University, Bangkok, Thailand; ^5^Centre for Tropical Medicine and Global Health, Nuffield Department of Medicine, University of Oxford, Oxford, United Kingdom; ^6^Cambodia Oxford Medical Research Unit, Angkor Hospital for Children, Siem Reap, Cambodia; ^7^Institute of Global Health, Faculty of Medicine, University of Geneva, Geneva, Switzerland

**Keywords:** neonatal care, evidence-based practice, newborn infant, premature birth, refugees, transients and migrants, developing countries (DC)

## Abstract

**Background:**

Preterm birth is a major public health concern with the largest burden of morbidity and mortality falling within low- and middle-income countries (LMIC).

**Materials and methods:**

This sequential explanatory mixed methods study was conducted in special care baby units (SCBUs) serving migrants and refugees along the Myanmar-Thailand border. It included a retrospective medical records review, qualitative interviews with mothers receiving care within SCBUs, and focus group discussions with health workers. Changes in neonatal mortality and four clinical outcomes were described. A mix of ethnographic phenomenology and implementation frameworks focused on cultural aspects, the lived experience of participants, and implementation outcomes related to SCBU care.

**Results:**

From 2008–2017, mortality was reduced by 68% and 53% in very (EGA 28–32 weeks) and moderate (EGA 33–36 weeks) preterm neonates, respectively. Median SCBU stay was longer in very compared to moderate preterm neonates: 35 (IQR 22, 48 days) vs. 10 days (IQR 5, 16). Duration of treatments was also longer in very preterm neonates: nasogastric feeding lasted 82% (IQR 74, 89) vs. 61% (IQR 40, 76) of the stay, and oxygen therapy was used a median of 14 (IQR 7, 27) vs. 2 (IQR 1, 6) days respectively. Nine interviews were conducted with mothers currently receiving care in the SCBU and four focus group discussions with a total of 27 local SCBU staff. Analysis corroborated quantitative analysis of newborn care services in this setting and incorporated pertinent implementation constructs including coverage, acceptability, appropriateness, feasibility, and fidelity. Coverage, acceptability, and appropriateness were often overlapping outcomes of interest highlighting financial issues prior to or while admitted to the SCBU and social issues and support systems adversely impacting SCBU stays. Interview and FGD findings highlight the barriers in this resource-limited setting as they impact the feasibility and fidelity of providing evidence-based SCBU care that often required adaptation to fit the financial and environmental constraints imposed by this setting.

**Discussion:**

This study provides an in-depth look at the nature of providing preterm neonatal interventions in a SCBU for a vulnerable population in a resource-limited setting. These findings support implementation of basic evidence-based interventions for preterm and newborn care globally, particularly in LMICs.

## 1. Introduction

Globally in 2019, mortality occurring within the first month of life claimed 2.4 million neonates' lives—nearly half of all deaths in children under 5 years of age (1). Within the first month of life, approximately 75% of all deaths occur in the first week (2, 3). Historically, nearly 99% of all neonatal deaths occur in low- and middle-income countries (LMICs) (4).

But this need not be the case. Evidence-based interventions for newborns can reduce mortality, morbidity, and long-term adverse outcomes associated with poor growth and development in neonates (5–9). Routine interventions can be provided within special care baby units (SCBU, i.e., without intensive neonatal care) and include the promotion of early and exclusive breastfeeding; adequate thermal care; hygienic cord care; early diagnosis of infections and early detection of respiratory distress syndrome (6, 9–11). The Lancet Every Newborn Study Group projects that interventions such as these implemented at 70–90% coverage by 2025 could avert over 70% of under five deaths (2, 6).

Among newborns, the mortality, morbidity, and long-term outcomes of preterm neonates (<37 weeks of gestation) are poorer than those born at term, with the shortest gestations carrying the highest risk of poor outcomes (12–15). An estimated 15 million neonates are born prematurely every year, and the majority are in LMICs where the social, economic, and life-long effects are often difficult to address (16–18). Prematurity carries risks of short-term respiratory, neurological, or digestive complications (15) and it is the leading cause of neonatal mortality (18–20). In addition to routine neonatal care interventions outlined above, standardized care for preterm newborns should also include antenatal, intrapartum, and neonatal care interventions for birth complications; neonatal resuscitation; therapeutic hypothermia; prevention and management of respiratory distress syndrome; and phototherapy treatment for neonatal jaundice (5, 7, 9).

Implementation of these evidence-based interventions for preterm newborns in LMICs remains low (1, 8). There have been calls for well-designed cohort studies from LMICs that define, measure, and report preterm birth data to ultimately improve quality of care and reduce mortality and morbidity in these settings (15, 27, 28). Published studies on improving the quality of newborn care in LMICs provide important insights into neonatal care provision in these settings, but focus predominantly on facility and service readiness, barriers and facilitators for service delivery, human resource constraints, and health worker capacity (4, 27–32). Few studies have been published on the implementation of newborn care in vulnerable communities such as those in humanitarian settings (33, 34). Increasing use of implementation frameworks may offer a more systematic evaluation of newborn care packages while clarifying equitable service delivery in vulnerable populations often present in LMIC settings (35, 36).

In a LMIC setting along the Myanmar-Thailand border, the Maternal and Child Health Department of the Shoklo Malaria Research Unit (SMRU) has aimed to reduce maternal and neonatal mortality for the past 38 years among refugees and migrants (37–40) through continuous quality improvement, featuring up-to-date guidelines and international standards of care adapted to this resource-limited setting (41). Facilities include structures necessary and sufficient for antenatal care provision, and labor and delivery wards equipped for basic emergency obstetric and newborn life support. SMRU facilities are staffed by locally trained medical staff (“medics“), nurses, midwives, sonographers, laboratory technicians, under the supervision of qualified doctors (42, 43). On-site staff have been trained in newborn resuscitation and participate in routine exercises based on the Basic Emergency Obstetric & Newborn Care Life Support (BEmONC^®^) curriculum (44). In 2008, SMRU established Special Baby Care Units (SCBUs) within its facilities for specialized care of preterm, small, or sick neonates (40); the first such unit for refugees and migrants outside the Thai hospital system. As in all other care units in SMRU facilities, the SCBU was set up to accommodate parents' presence near their newborn. The establishment of the SCBU offset the feelings of helplessness that SMRU doctors, medics, nurses, and parents felt with preterm births. With growing confidence and practice, these SCBU services showed that preterm, small, and sick newborns could be cared for in this LMIC setting (40). However, new constraints and barriers were soon perceived, in particular when caring for the younger, sicker, and smaller neonates.

As part of this continuing process to improve maternal and newborn care delivered at SMRU and to add to the literature on newborn care delivered in LMIC settings, this sequential explanatory mixed methods study reviews 10 years of SCBU care provided to refugee and migrant newborns along the Myanmar-Thailand border. It summarizes mortality trends and clinical outcomes among preterm neonates regarding four main points of care: feeding, infections, thermal care, and respiratory care. In addition to these clinical outcomes, this study incorporates key implementation research outcomes for SCBU care provision in this setting.

## 2. Materials and methods

This study employed a sequential explanatory mixed methods design. Decision to perform the chart review up to the end of 2017 was due to two major changes in the provision of health care along the Myanmar-Thailand border occurring over this period: preventive and curative health care services provided by SMRU in Mae La refugee camp was transferred to a single non-governmental organization in early 2018. Secondly, beginning in September 2017, a social enterprise launched a low-cost, non-profit health access fund for migrants along the Myanmar-Thailand border to support health-related costs. This program allowed pregnant women attending SMRU ANC clinics easier access to tertiary hospital care in the case of obstetric complications including those related to preterm labor (45–47). Therefore, to control for these externalities affecting the migrant and refugee communities relating to SCBU service provision, medical charts completed prior to the end of 2017 were included in this study.

### 2.1. Study setting and participants

Data on refugees came from Mae La camp, which had an estimated population of 37,786 refugees in 2016 (48). SMRU provided ANC services to approximately 90% of the women in the Mae La camp with 75% of them delivering at the SMRU clinic (25, 37, 49–51). SMRU also serves the migrant communities within Tak province, Thailand, and Karen State, Myanmar, through clinics in Maw Ker Thai and Wang Pha, Thailand. The estimated catchment of these clinics is approximately 200,000 migrants (52). The predominant ethnic group along the border during the study period was the Karen, but the flow of migrants from neighboring Myanmar has led to a greater diversity of communities served by SMRU. Both refugee and migrant communities come from socially and economically disadvantaged regions in Myanmar with poor access to maternal and newborn health services (53–55).

### 2.2. Special care baby unit practices and protocols

Accurate estimation of gestational age has always been an integral part of the antenatal care and since 2001 dating is based on ultrasound (37–40). SMRU provides facility-based, routine and specialized services for antenatal, intrapartum, delivery, postpartum, and newborn care. Maternal and newborn care has been provided to refugees beginning in 1984 and expanded coverage to include migrant populations beginning in 1998. Preterm birth rates have been relatively stable between 7 and 10% annually (SMRU annual reports). The largest proportion of preterm births (85%) occur within 32–37 weeks of gestation; most women deliver in one of the SMRU delivery suites (60%) however nearly 20% of preterm births still occur at home.

Specific care for preterm, small, and sick newborns has evolved over the years and includes evidence-based interventions provided by SMRU from preconception through the newborn period (Table 1). Prior to 2008, midwives provided medical care to all newborns in the postnatal ward; using minimal equipment (resuscitation self-inflating bag and mask, nasogastric feeding, intramuscular antibiotics) and followed treatment recommendations of doctors. A fatalistic attitude toward survival of preterm, small, and sick newborns from both families and medical staff was common and parents would often choose to take their newborns back home. While infant mortality was drastically reduced from 183 to 78 deaths per 1,000 livebirths between 1987 and 1996 after recognition of infantile beriberi and routine B1 administration during pregnancy (38), neonatal mortality decreased less rapidly in subsequent years (30.3 in 1991–1995 to 24.4 deaths/1,000 livebirths in 1996–2000).

**Table 1:** Provision and coverage of evidence-based interventions provided as part of routine care at SMRU clinics for refugees and migrants, adapted from Vaivada et al. (9).

**Effect**	**Preconception and pregnancy**	**Labor, birth, newborn**	**Interventions from preconception to newborn care**
Child mortality	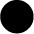 Routine antenatal care, management of maternal chronic illness and pregnancy complications 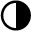 Maternal nutritional supplementation^a^	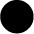 Skilled birth attendance 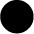 Clean birth kits 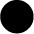 Delayed cord clamping and hygienic cord care 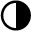 Emergency management of birth complications and asphyxia 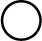 Kangaroo mother care for low birthweight neonates^b^ 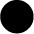 Corticosteroids for imminent preterm birth, specialized care for preterm, low birthweight, and ill neonates 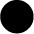 Promotion of early initiation and exclusive breastfeeding	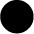 Routine age-appropriate vaccination for mothers 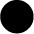 Routine age-appropriate vaccination for infants^c^ 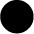 Provision and promotion of insecticide-treated bednets 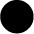 Antibiotic treatment for severe infections and sepsis
Child morbidity	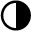 Lifestyle interventions for gestational diabetes mellitus^d^ 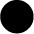 Antenatal infection screening and treatment	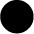 Maternal antibiotics for prolonged premature rupture of membranes 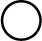 Kangaroo mother care for healthy newborns^b^ 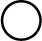 Topical emollient therapy for preterm neonates 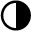 Ibuprofen for patent ductus arteriosus^e^	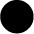 Promotion of improved water, sanitation, and hygiene conditions
Nutrition, growth, development	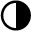 Periconceptional folic acid^f^ 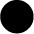 Small quantity lipid-nutrient supplements 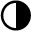 Support for maternal mental health	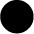 Magnesium sulfate for fetal neuroprotection and early development intervention for preterm infants^g^ 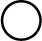 Sound reduction for preterm in NICUs 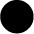 Prophylactic phototherapy for preterm birth and low birthweight neonates with jaundice	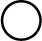 Prevention of gender-based violence

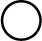
 = little to no coverage; 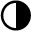 = partial coverage; 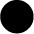 = full coverage. ^a^Provided as micronutrient fortified foods and food supplements during antenatal care visits (21). ^b^Culturally adapted swaddling (22). ^c^Facility-based vaccination program in place and provides infant immunizations, but poor follow up restricts coverage rates in the first year of life (23, 24). ^d^Risk factor-based screening for gestational diabetes mellitus with treatment over the antenatal period (25). ^e^Not all murmurs are routinely checked with cardiac ultrasound. ^f^Periconceptional folic acid coverage is low (<2%) (26). ^g^Given to mothers at risk of pre-eclampsia.

The SCBUs established in 2008 follow the basic concept of “MACHO”: M, Milk; A, Antibiotics; C, Cord care; H, Heat; O, Oxygen. Newborn care provided according to MACHO was implemented to guide the locally trained medical staff in providing systematic care to all neonates and developing appropriate management plans. Locally appropriate and standardized neonatal guidelines were developed at the same time, based on the WHO integrated management of childhood illness and relevant published literature on feeding, infections, thermal care, and respiratory care. These guidelines are reviewed and updated regularly within SMRU by doctors and local staff.

Maternal milk is the main source of milk and donor milk is sought as a second option; formula is rarely offered and avoided for neonates born <34 weeks of gestation. Preterm formula and human milk fortifiers are not available. Systematic screening for Group B streptococcus in pregnancy is not proposed, although carrier status in this population is not uncommon (12%) (56). Bacteriologically-confirmed neonatal sepsis is rarely achieved in this setting (4.5% of blood cultures with at least one organism of significance or possible significance) and therefore the decision to start intravenous antibiotics is based on clinical signs and risk factors (51). Neonates are kept warm in an infant radiant warmer or on a warm blanket, swaddled and surrounded by warm water bottles; the concept of skin-to-skin contact is not well perceived by the population who has a strong traditional belief in swaddling the newborn immediately after birth (22). In total of 100%, oxygen (delivered *via* mask or nasal cannula) is provided to neonates with signs of severe respiratory distress, transient tachypnea or respiratory distress syndrome in order to maintain their oxygen saturation above 90%. Blended oxygen, continuous positive air pressure and surfactant are not available.

Neonatal hyperbilirubinemia (57–59) is treated by phototherapy following the British NICE guidelines (60). Exchange transfusion is possible in Mae Sot General Hospital in Tak Province, Thailand.

All inpatients and caregivers are provided with meals twice per day at no cost.

### 2.3. Study design

Quantitative analysis was performed by retrospective chart review of all neonates born prematurely (<37 weeks of gestation) to mothers attending antenatal SMRU clinics between January 2008 and December 2017 and for whom a medical chart was available. Qualitative components included focus group discussions (FGDs) with medical staff working in the SCBU (June 2020) and interviews with mothers of preterm, sick, or small neonates receiving SCBU care (June 2020 to March 2021). Medical staff included medics and nurses with work experience in the SCBU who provided written informed consent. Mothers included those aged 18 years and above who provided informed consent to be interviewed.

### 2.4. Chart review

Information contained in the medical records is routinely coded by the medical staff prior to data entry and includes: a unique identification number; basic demographic characteristics; anthropometric measurements; summary of clinical findings; and final possible diagnosis. Additional information related to morbidity-specific conditions of interest was extracted from the medical records by medical staff and doctors with SCBU experience. We excluded neonates born and hospitalized in a tertiary hospital, those born at home and not subsequently hospitalized in SMRU clinics, those born in SMRU clinics but transferred to tertiary hospitals for further care, or those receiving palliative care (61). Data extraction was performed by following a pre-coded form and then entered to an electronic database by unique identification number.

#### 2.4.1. Outcomes of interest

Primary outcomes of interest were neonatal mortality and morbidity of preterm neonates <37 weeks of gestation. Mortality was stratified according to severity of prematurity: extreme (EGA < 28 weeks), very (EGA 28–32 completed weeks), and moderate (33–36 completed weeks). Timing of death (0–7 days, 8–28 days or beyond the neonatal period) was analyzed over time and tested for reduction in mortality rates over the duration of SCBU establishment. These three periods include (1) the early, “establishment” period between 2008 and 2011 when SCBUs were adapted, piloted, and introduced to the SMRU care system; (2) an “expansion” period between 2012 and 2014 when SCBU interventions and care were established at all SMRU clinics; and (3) a “routine” period where SCBU care was provided routinely at all SMRU sites (2015–2017). The transition between each of these periods allowed for review, process evaluations, and continuous quality improvement for SCBU and newborn care packages provided at SMRU.

Other outcomes of interest related to morbidity were temperature regulation, feeding, respiratory support, and infections during the stay in SCBU.

#### 2.4.2. Clinical outcomes analysis

Statistical analysis was performed using Stata Statistical Software (*StataCorp*. 2021. *Stata* Statistical Software: Release *17*). Descriptive data were presented using proportion, mean and standard deviation or median and interquartile range as appropriate. Differences in prematurity groups or periods were compared by chi-square test or Kruskal-Wallis rank test. Percentage change in mortality within prematurity groups was calculated using the formula:

Percent change in mortality = [(mortality in 2015–2017 – mortality in 2008–2011)/mortality in 2008–2011]^*^100.

Incidence rates for early neonatal deaths (0–7 days), later neonatal deaths (8–20, 27–34) and infant deaths were calculated for each prematurity groups and Kaplan-Meyer curves created. SCBU stay duration and secondary outcomes were evaluated from medical charts of neonates who survived the early neonatal period, and for whom data were available from the first 24h of life.

### 2.5. Qualitative methods and analysis

FGD and interview guides were developed by VC and AH based on literature review of SCBU best practices. Guides focused on barriers and facilitators for common treatment procedures including early onset neonatal sepsis and thermoregulation; apnea and supportive respiratory care; feeding and growth; and jaundice.

The qualitative approach employed was a mix of ethnography and phenomenology focused on cultural aspects as well as the lived experience of participants in receiving or providing SCBU care. Hence, a constructivist paradigm was used.

The qualitative research team was made up of VC, AH, KKA, DM, and PM, with two clinical researchers (VC and AH) and two social science researchers (AH and PM) with a combined experience of multiple qualitative studies. These researchers were not Karen or Burman but collectively had much research experience with these two ethnic groups. Two members of the research team (KKA and DM) were ethnically Karen and fluent in Burmese, and collectively participated in facilitating and conducting qualitative interviews in this context (among refugees and migrants) for multiple studies conducted at SMRU. Their primary role was facilitators and were involved in analysis to ensure fidelity to the voices of participants. These members were from the communities that medical staff and patients come from, ethnically Karen, and conducted interviews in Karen and Burmese based on the participant's preferred language. FGDs and interviews were led by KKA, and no research staff participating in FGD or interviews had clinical duties or involvement in direct patient care. Focus group discussions and interviews were conducted with at least one other supporting staff present (AH, PM, and DM) for help facilitating and notetaking. All staff had been trained in qualitative methods and trained specifically for this study by AH and VC.

FGDs and interviews were audio-recorded for later transcription and translation into English. Immediately following FGDs and interviews, study personnel (VC, AH, KKA, DM, and PM) would debrief to identify the main themes emerging from each discussion with KKA reviewing the recording and compiling notes. Preliminary findings from FGDs were reviewed with medical staff for confirmation. KKA and AH determined when saturation had been reached for FGD and interviews, respectively. Translation involved AH, KKA, DM, and an outside translator, ethnically Karen, fluent in both Karen and Burmese, and a refugee. The first 3 transcriptions (1 FGD and 2 interviews) were performed by AH, KKA, DM, and the outside translator, to demonstrate the clarity and depth of translation needed. The remaining translations were then performed by KKA and the outside translator to ensure the quality and comprehensiveness of transcriptions, with KKA and AH clarifying difficulties in translation by referring to notes and shared experience of facilitating FGDs and interviews.

FGD and interview data were combined for analysis and AH, KKA, PM, and VC performed preliminary inductive analysis to identify key themes related to barriers and facilitators to SCBU care. Codebooks were finalized once consensus was reached. This process ensured triangulation of key findings, and by involving those directly involved in conducting FGDs and interviews, provided fidelity in reporting participant voices. AH applied the Implementation Outcomes framework (Table 2) (36) and, where aligned, the EquIR framework (35) to the identified themes. “Intervention” in this study was defined as being admitted to the SCBU and/or receiving specific evidence-based treatment according to guidelines (e.g., early onset neonatal sepsis). Implementation frameworks offered a more systematic evaluation of the program using the qualitative data while clarifying equitable service delivery in this vulnerable population (i.e., refugees and migrants). Implementation outcomes of interest included: acceptability, appropriateness, coverage, feasibility, and fidelity. Qualitative methods therefore corroborated quantitative analysis summarizing clinical effectiveness and added the lived experience of providing care in this setting according to implementation outcome frameworks.

**Table 2:** Implementation outcomes [IO; (36)] and EquIR (35) frameworks for thematic analysis of qualitative components.

**Implementation outcomes**	**Definition**
Acceptability	IO: The perception among implementation stakeholders that a given treatment, service, practice, or innovation is agreeable, palatable, or satisfactory.
Appropriateness	IO: The perceived fit, relevance, or compatibility of the innovation or evidence-based practice for a given practice setting, provider, or consumer; and/or perceived fit of the innovation to address a particular issue or problem.
Coverage	EquIR: The degree of reach, access, service spread or effective coverage for the disadvantaged target population.
Feasibility	EquIR: The extent to which a program reduces barriers and can be carried out in disadvantaged population.
Fidelity	IO: Comparing the original evidence-based intervention and the disseminated/implemented intervention in terms of (1) adherence to the program protocol, (2) dose or amount of program delivered, and (3) quality of program delivery.EquIR: Adherence of disadvantaged populations to equity-focused implementation of intervention.

## 3. Results

### 3.1. Retrospective chart review

Between January 1, 2008 and December 31, 2017, a total of 2,319 preterm births were documented, of which, 203 stillbirths were excluded from further analysis. Annually, there was an average 230 preterm births (IQR 222, 264), ranging from a maximum of 286 in 2010 to a minimum of 122 in 2017. The proportion of preterm birth among all live births from women attending antenatal care clinics at SMRU varied between 10.5% in 2008 and 7.4% in 2017.

#### 3.1.1. Mortality

**Figure 1:**
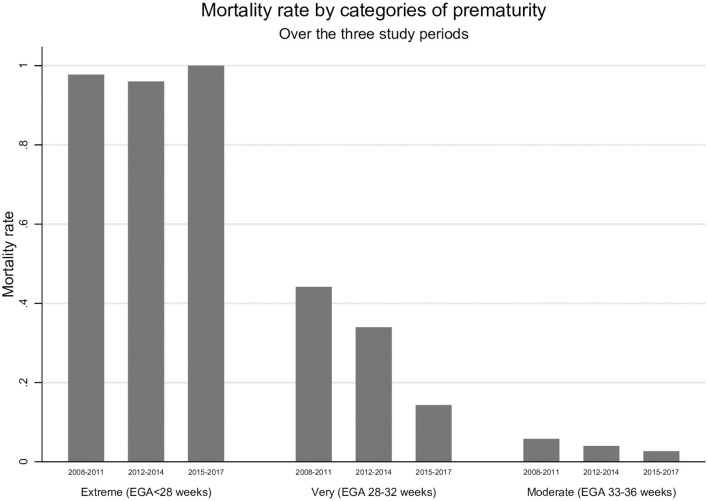
Mortality rate by categories of prematurity and by study periods.

**Figure 2:**
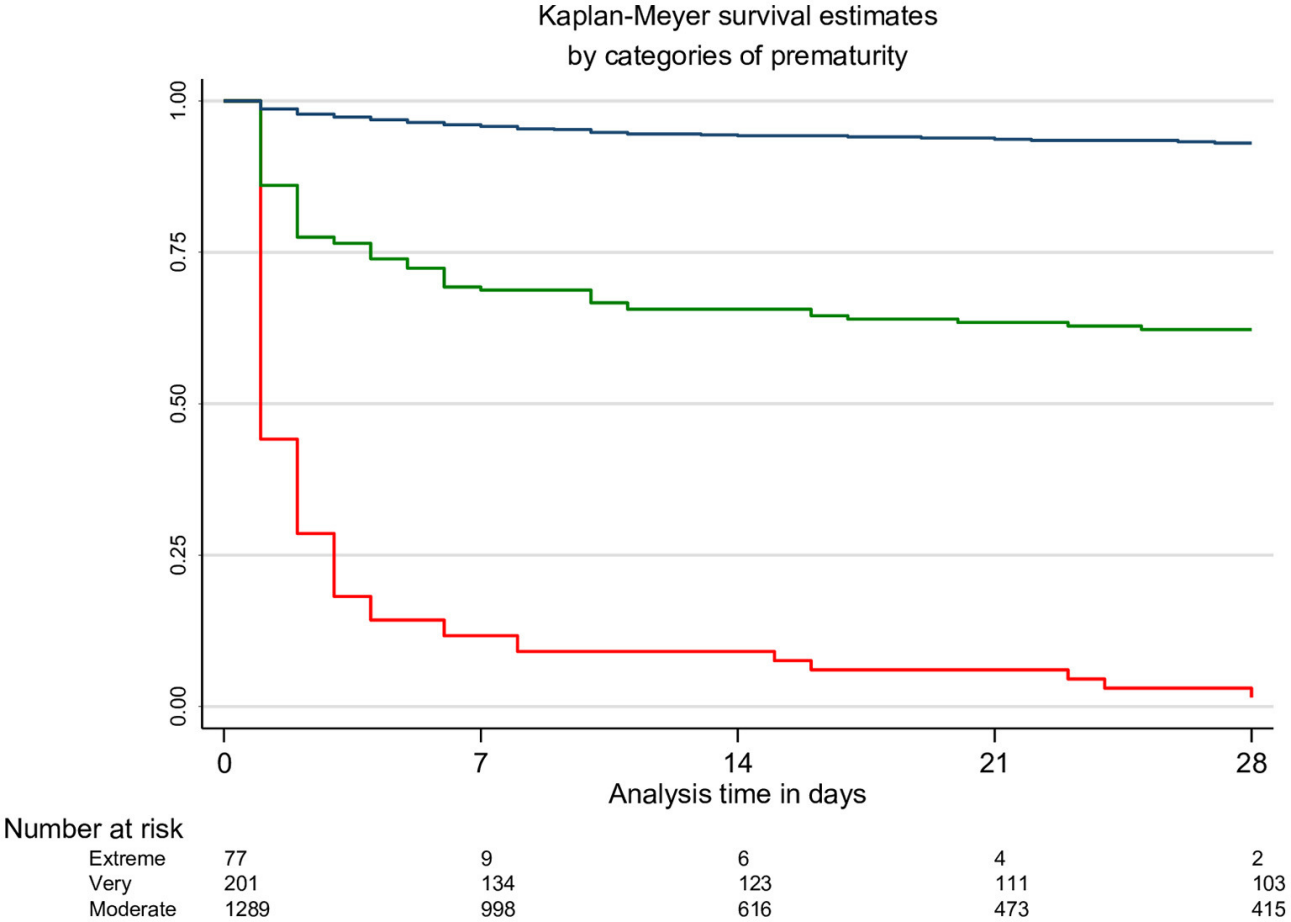
Kaplan-Meyer survival estimates and number of neonates at risk table, by categories of prematurity. Red line: extreme preterm neonates (EGA < 28 weeks); green line: very preterm neonates (EGA 28–32 weeks); blue line: moderate preterm neonates (EGA 26–33 weeks).

There were 235 deaths documented over the study period, with the proportion of deaths decreasing significantly from 14.4% (144/999) during the early establishment period of SCBU (2008–2011) to 10.5% (63/603) during the expansion of SCBU care to all sites (2012–2014) and to 5.5% (28/514) while the continuum of care was performed more routinely at all sites (2015–2017) (*p* < 0.001). The largest mortality reduction (68%) was observed among very preterm neonates (EGA 28–32 weeks). Mortality was reduced by 53% in moderate preterm neonates (EGA 33–36 weeks), while no change was observed among the extreme preterm (EGA < 28 weeks) (Figure 1). Most deaths, 77% (181/235), occurred in the early neonatal period, of which half were within hours of birth (88/181), with a mortality incidence rate of 382.0 [95%CI 301.2–484.5], 56.8 [95%CI 44.3–72.9], and 6.4 [95%CI 4.8–8.4] deaths per 1,000 neonate-day in extreme preterm, very preterm and moderate preterm neonates respectively (Figure 2 and Supplementary Table 1). Among the 16 neonates (EGA 29 + 2 to EGA 36 + 6) who died after the neonatal period, 9 died between day 30 and 56 of life, and the remaining 7 died between day 127 and 345 of life. Five died of complications due to severe congenital abnormality, two because of an acute severe infection, and there was one accidental death. Cause of death was unknown for the remaining 8 neonates.

#### 3.1.2. SCBU stay duration

A total of 1,358 medical charts were available and 963 were evaluated that corresponded to the inclusion criteria mentioned in the methods.

Duration of SCBU stay ranged from 1 to 82 days with a median of 11 days (IQR: 5, 19 days). This duration increased by 1 day over the 3 SCBU periods: 10 days (IQR 3, 16) in 2008–2011, 11 days (IQR 6,19) in 2012–2014, and 11 days (IQR 7, 21) in 2015–2017, *p* = 0.001. Very preterm neonates (EGA 28–32 weeks) discharged alive from the SCBU remained under observation for nearly 3 weeks longer than moderate preterms (EGA 33–36 weeks), with a median of 35 days (IQR 22, 48 days) compared to a median of 10 days (IQR 5, 16). Neonates' characteristics upon admission to SCBU care are presented in Table 3.

**Table 3:** Characteristics of 963 preterm neonates upon admission to the SMRU SCBU (EGA: estimated gestational age).

	**Overall**	**Very preterm (EGA 28–32 weeks)**	**Moderate preterm (EGA 33–36 weeks)**
Mean weight in kg (SD) (*n* = 962)	2.09 (0.46)	1.41 (0.27)	2.17 (0.40)
Hypothermia (temp < 36.5°C)	345/950 (36.3%)	58/103 (56.3%)	287/847 (33.9%)
Abnormal respiratory rate (>60 breaths/minute)	208/953 (21.8%)	48/104 (43.8%)	160/849 (18.9%)
On oxygen	218/956 (22.8%)	64/104 (61.5%)	154/852 (18.1%)
Transient tachypnoea of prematurity	40/963 (4.2%)	12/104 (11.5%)	28/859 (3.3%)
Antibiotics IV	348/957 (36.4%)	82/104 (78.9%)	266/853 (31.2%)
**Feeding type**			
Breast milk	660/957 (69.0%)	29/104 (27.9%)	631/853 (74.0%)
Donor milk	37/957 (3.9%)	7/104 (6.7%)	30/853 (3.5%)
Powder milk	28/957 (2.9%)	2/104 (1.9%)	26/853 (3.1%)
A mix of above milk types	32/957 (3.3%)	1/104 (1.0%)	31/853 (3.6%)
Nil by mouth	200/957 (20.9%)	65/104 (62.5%)	135/853 (15.8%)

#### 3.1.3. Thermal care

Hypothermia (temperature < 36.5°C) was the most common cause of abnormal body temperature on admission, with a median temperature of 36°C (IQR 35.7, 36.2°C). Eighty neonates had a fever on admission, a median of 37.9°C (IQR 37.7, 38.1°C) with a maximum body temperature of 39.0°C. The return to and maintaining an optimal body temperature were challenging, with neonates spending a median 5 days (IQR 1, 12) with body temperatures outside the normal range.

#### 3.1.4. Infections

More than one-third of neonates (36.4%, 348/957) received intravenous antibiotics on admission to SCBU based on the clinical algorithms in place. As expected, febrile neonates were more likely to receive IV antibiotics (71,3%, 57/80), while 40.9% of hypothermic neonates (131/320) received IV antibiotics.

Diagnosis of meningitis was made in only 2 neonates at 1 and 4 days of life—one confirmed with high white blood cells count in the lumbar puncture. Sepsis on the other hand was frequently clinically suspected (23.3%, 224/963), significantly more in very preterm neonates (48.1%, 50/104) compared to 20.3% (174/859) in moderate pre terms (*p* < 0.001). As per guidelines, these neonates received a 5 or 7-day IV antibiotic course, occasionally extended to 10 days. Cord infection was rare (2.5%, 24/963) and treated with oral or IV antibiotics depending on the clinical condition.

#### 3.1.5. Feeding

In neonates with daily weights available, time needed to return to birth weight was a median 6.5 days (IQR 2, 12.5) and 6 days (IQR 2, 10) for very and moderate pre terms respectively, after which they gained 17 grams (IQR 13, 21) and 15 grams (IQR 6, 26) daily until discharge, respectively. On admission, a large majority of neonates (79.1%, 757/957) were already fed with: maternal breast milk (69.0%), donor milk (3.9%), powder milk (2.9%), or a combination of milk (3.3%). The remaining neonates (20.9%, 200/957) received IV fluid. Feeding with nasogastric tube (NGT) was commonly used: 96.2% (100/104) and 52.4% (450/859) of very and moderate preterm neonates received feeding *via* NGT for a duration of 26 days (IQR 17.5, 38) and 9 days (IQR 4, 14) respectively, or 82% (IQR 74, 89) and 61% (IQR 40, 76) of their stay in the SCBU. Nearly all the mothers (93.2%, 513/550) expressed milk manually for each feed for the full duration of the NGT feeding.

Complications related to feeding were frequent over the course of SCBU stays: 13% (129/963) of neonates had one episode of hypoglycemia and 2% (20/963) had 2 or more episodes recorded. Eleven percent of neonates vomited their feed (107/963), ranging from one to 12 episodes each, and 8.7% (84/963) had severe abdominal distension. Despite these feeding difficulties, stopping or missing feeds were reported for only 12 neonates (all moderate pre terms).

Blood in the stools was rarely documented (in 3 very and 3 moderate pre terms); it lasted 1–5 days, was associated with abdominal distension in 5 neonates, vomiting in 4, and in 3 instances neonates were treated with IV antibiotics and oral feeding was suspended for suspected necrotizing enterocolitis.

#### 3.1.6. Respiratory care

Higher than expected respiratory rates [median rate of 68 (IQR 64, 74, maximum 110)] were found in 208 neonates (21.8%, 208/953) and 3 neonates had a low respiratory rate (<30/min) on admission. Transient tachypnoea of the newborn (TTN) was reported in 40 neonatal charts (4.2%), significantly more often in very than in moderate preterm neonates (11.1%, 12/104 vs. 3.3%, 28/859, *p* < 0.001). All but one very preterm and 71.4% (208) of moderate preterm neonates with TTN received oxygen, with a median respiratory rate of 70 (IQR 64, 76) and maximum of 110. Most were kept nil by mouth (60.0%, 24/40) and recovered within 24–48 h.

In addition to the 218 neonates receiving oxygen upon admission, 56 (18 very and 38 moderate pre terms) required oxygen during the course of the hospitalization. The overall duration of oxygen therapy was 14 days (IQR 7, 27) and 2 days (IQR 1, 6) in very and moderate preterm neonates, respectively. Overall, respiratory distress was reported for 24.8% of neonates (239/963) ranging from 1 to 66 days, with apnea being the main concern and reported in 52.9% (55/104) and 5.8% (50/859) of very and moderate preterm neonates for a median of 2 days (IQR 1, 3), maximum 20 days and 1 day (IQR 1, 2), maximum 13 days respectively. Clinical diagnosis of pneumonia was however rarely reported [2.3% (22/963)].

### 3.2. Qualitative results

A total of 4 FGDs with 27 medical staff were conducted including 2 FGDs with 13 medics (9 men and 4 women) and 2 FGDs with nurses (3 men and 11 women). Medical staff averaged 6.9 +/– 3.1 years of experience working at SMRU (range: 2 to 13 years).

A total of 9 interviews with mothers were completed: 2 had term neonates, seven had late (*n* = 3), moderate (*n* = 2) and very (*n* = 2) preterm neonates. The average age of mothers was 33.4 +/– 9.9 years (range: 20 to 45 years), 4/9 (44.4%) were of Karen and 5/9 (55.6%) of Burman ethnicity, and all but one mother was multiparous. Newborn EGA was 34 weeks + 6 days +/– 2 weeks + 6 days (range: 30 weeks + 4 days to 37 weeks + 6 days). There were 5 neonates with jaundice and 4 were <2,500 grams.

Mothers of neonates admitted to the SMRU SCBU explained acceptability, appropriateness, and coverage outcomes related to SCBU care. FGDs with medical staff explained feasibility, fidelity, and effectiveness outcomes. Both mothers and medical staff explained outcomes related to appropriateness. Results suggest that SCBU care coverage, acceptability, and appropriateness, were more closely related to patient outcomes. Once coverage, acceptability, and appropriateness were addressed, feasibility and fidelity of implementing evidence-based SCBU care to this population were highlighted as secondary but important themes.

#### 3.2.1. Coverage, acceptability, appropriateness

Coverage, acceptability, and appropriateness were often overlapping implementation outcomes of interest explained by mothers' experiences accessing clinics and SCBU care, financial issues prior to or while admitted to the SCBU, and social issues and support systems as they affected SCBU care.

Coverage of SCBU care—the degree of reach and access mothers had to SCBU facilities—takes logical precedent over other related outcomes. All mothers interviewed provided their experiences, corroborated by medical staff, of the often-fraught journey to find quality antenatal, birthing, and SCBU care that limited the coverage of services to this population. All interviewees reported that access to SCBU facilities was constrained, and the majority reported a mix of factors including poor infrastructure in the cross-border region, associated financial burdens, lengthy travel to clinic sites, and undocumented migrant status.

“Our village is very difficult to reach by transportation and when I started having very bad delivery pain, I phoned someone I know who lives in the town near us to take me to the clinic. But I gave birth at home before he arrived here […]. Later some of the SMRU staff I know told me that my child is premature so I need to go to the clinic but I wasn't able to come here immediately. So, I came for follow up when my child was already 10 days old. My child had a bit of jaundice.”—Mother (≥35 years), interview, SMRU Maw Ker Thai Clinic (MKT-IDI-06).

All women reported a willingness to travel great lengths and undertake great risks in seeking the SCBU services provided by SMRU clinics, best expressed in this next dialogue between a mother and the interviewer:

Mother: “I cannot speak Thai so I never go to the Thai hospital and only come here. I have never gone to the Thai hospital in either Mae Ra Mat or Mae Sot [Thailand].”

Interviewer: “Is this closer for you?”

M: “Yes, this is the closest for me to get to. There is another hospital, but I don't go there.”

I: “How long does it take for you to travel from your village to get here?”

M: “It takes one and half hours by motorbike. But if I come by foot then it can take me 2 or 3 h. At the foot of the hill, there is a Thai police check point. We need to be careful when coming from the forest.”

I: “Then, what difficulties do you have to come for ANC follow up?”

M: “The difficulty is to come by motorbike. Sometimes, I have money for that but sometimes I do not. We need to fix the motorbike very often. Sometimes, we can come but sometimes cannot. When I first came for ANC follow up, I was only 7 weeks pregnant and I only came 3 times [over the course of my pregnancy].”—Mother (21–34 years old), interview, SMRU Wang Pha clinic (WPA-IDI-01).

However, as reported by 7 out of the 9 mothers interviewed, financial burdens, social issues related to home life, or both were a reason to want to leave the hospital. These external issues limited the acceptability and appropriateness women felt in accessing clinics or being admitted to the SCBU facility for long periods of time. Take this mother's explanation for example:

“The only [issue being here in the SCBU] is that not being at home is very different. At home, I need to manage food for the family by talking with the family and arranging it for them. If I am at home, I can be frugal and not waste any money. If not, they will use a lot more money than they should. On one hand, I worry for the family and also being here away from the family can cost money, too. Sometimes, if I can't make it for mealtime [at the clinic] then I need to buy some food outside the clinic. Because of that, money keeps running out. So, I want to go back home.” —Mother (≥35 years old), interview, SMRU Maw Ker Thai Clinic (MKT-IDI-06).

Despite these difficult circumstances around access and financial issues, the majority of the women interviewed (*n* = 8) realized the need for SCBU care for their newborns, especially given the positive interaction with the medical staff who helped to explain their neonate's situation and rationale for intensive treatment. Indeed, medical staff compassionate care and communication with mothers were often cited as enhancing the acceptability and appropriateness with intensive treatments. The patient-centered care—culturally sensitive and provided in the women's native tongues—offset risks associated with both seeking care and the substantial toll that extended hospital stays took on women's personal lives.

“I'm not angry with [the medical staff]. Because they do their best for my baby. But sometimes I feel sad to see my baby in pain. However, I feel OK with that because if I am at home I can do nothing if my baby feels unwell. Sometimes, the staff scold us but I know that they want the best for our children.”—Mother (≥35 years old), interview, SMRU Maw Ker Thai Clinic (MKT-IDI-01).

“Some mothers tell us that they pity their baby. We can only respond that we pity the baby as well. However, we are not doing bad for your baby but doing the best for your baby. Then we need to explain to them again and again that even though we are staff at the clinic, we pity the baby, too. But we want the best for the baby so we have to do what we are doing now.”—Nurse (4–12 years SMRU experience), FGD, SMRU Maw Ker Thai Clinic (MKT-FGD-02).

Quotes such as these demonstrate the lengths medical staff went to impress upon mothers the need for SCBU services, implying that staff viewed SCBU services as acceptable and appropriate for this population. Indeed, acceptability and appropriateness of SCBU services was almost never questioned by medical staff and were predominantly viewed as necessary for improving neonatal outcomes in this vulnerable population.

#### 3.2.2. Feasibility and fidelity

Interview and FGD findings highlight the barriers in this resource-limited setting as they impact the feasibility and fidelity of providing evidence-based SCBU care. Medical staff in all FGDs mentioned the need to adapt interventions to fit the financial and environmental constraints imposed by this setting. These limitations were reflected in both the feasibility of carrying out SCBU protocols and maintaining fidelity to the treatments as prescribed.

The most obvious determinant of fidelity relates to the need for well-functioning equipment required for high quality care—mentioned in all FGDs. Equipment issues related to all aspects of SCBU treatments involving respiratory support, phototherapy, feeding, and thermoregulation. Some exemplary quotes are included in Table 4.

**Table 4:** Exemplary quotes from focus group discussions with medical staff highlighting resource-poor setting effects on feasibility and fidelity of evidence-based SCBU treatments.

**Type of SCBU care**	**Quotes**	**Themes**
Feeding	“Well, we can help the patients but it takes more effort for the staff to feed them because they have to be fed hourly. Some of the patients are not severe, but the staff need to help them because the parents don't know how to. Sometimes premature infants need donor milk because they [need to supplement breastfeeding]. For donor milk, we need to find from breastfeeding mothers in the SCBU. Some mothers want to give but others do not. Even some mothers want to give, we have to screen their blood before we take milk. Also, we get some donor milk from Mae Sot.”—Medic (2–13 years SMRU experience), FGD, SMRU Maw Ker Thai Clinic (MKT-FGD-01).	Feasibility
Hyperbilirubinemia	“Some of the pre terms with jaundice need phototherapy. There is an eye mask to cover the baby's eyes. But there are not enough and sometimes they are not of good quality. We need to tie it to fit the baby because it's too big. There are not a lot of size M or size S to order for the baby. So, it is not ok for preterms. And many things from here were sent to Ko Ko SCBU [new SCBU unit on the Myanmar side], so here there is not really enough equipment. It is really difficult for us.”—Nurse (2–6 years SMRU experience), FGD, SMRU Wang Pha Clinic (WPA-FGD-02).	Feasibility
Respiratory support	“If you have a small oxygen face mask then it will be better. Also, [having an] oxygen flow meter which is 0.5 L [oxygen flow meter helps determine the flow of oxygen to the patient]. Now, we have one which is 15 L and if we open [it is difficult to control and fine tune]. It will be better if they can procure this for us. Because the preterms need oxygen support for long periods of time so it will be better to have 0.1 or 0.5 unit for that. At the moment, we have only one.”—Medic (2–10 years SMRU experience), FGD, SMRU Wang Pha Clinic (WPA-FGD-01).	Fidelity
Thermoregulation (e.g., early onset neonatal sepsis)	“When the pre terms have a cold temperature, then we use the incubator to warm them. But if the machine is not working properly or we don't have one available then we need to use a hot water bag. Then we need to be really careful about it. If not, it is very easy to burn the baby's skin. I think the incubator is better than the hot water bag. It will be better if we can have enough incubators for preterms who have low body temperature.”—Medic (2–10 years SMRU experience), FGD, SMRU Wang Pha Clinic (WPA-FGD-01).	Feasibility, fidelity

All discussions with medical staff revealed that evidence-based guidelines required adaptation in this setting, where the border context greatly differs from the clinical settings in high-income countries that generate much of the evidence for SCBU care. Medics from two FGDs reported that this clinical setting presented issues that may lead to confusion around performing SCBU interventions as prescribed.

“In the previous guideline, if the preterm newborns have fever then we need to treat them for the whole week. We do [as it is in the guidelines]. But sometimes, some of the doctors come during [early onset neonatal sepsis] treatment and if they notice the preterm newborns have no fever then they decide to do a CBC, CRP [blood tests for complete blood count and C-reactive protein (to test for inflammation or infection)]. If the results are good then they stop treatment before reaching a whole week. So, if you will update the guideline in the future, please add more details about what to do exactly, when we can stop, etc.”—Medic (2–10 years SMRU experience), FGD, SMRU Wang Pha Clinic (PCS-WPA-FGD-01).

“Another thing is [frequently monitoring] newborns receiving phototherapy. For example, here we must adjust by ourselves based on the frequency. So, that becomes another disagreement among us. Therefore, it would be better if we can have the exact guideline of when and how often to check, when to turn [phototherapy lights] off and what to do if the baby becomes very hot, etc. As one of the [doctor/consultant] said, if we get a new checklist, it says 72 hours. It is 3 full days. What about this season? Is that for every 6 hours or every 12 hours? I would like to ask the doctors to make detailed instructions for management in the new guidelines.”—Medic (2–10 years SMRU experience), FGD, SMRU Wang Pha Clinic (PCS-WPA-FGD-01).

## 4. Discussion

This study provides valuable insight on several intersecting topics: implementing evidence-based interventions to improve newborn health among refugees and migrants in a dynamic situation on the border between two LMICs. This mixed methods study conducted in this border context demonstrates the positive impacts of the introduction, improvement, and maintenance of facility-based neonatal care services in reducing newborn mortality and morbidity. It also provides key stakeholder perspectives—patients and providers—that help to characterize and suggest outcomes that may be considered for implementing newborn health services in resource-limited settings.

This study also serves as a means to guide and improve newborn care in this and other LMIC settings. This study shows how newborn care interventions are “fit” to this setting, taking from evidence generated elsewhere in the world—often from high-income settings (9).

The creation of a space dedicated to neonatal care, with access to simple but specific neonatal equipment, and services provided by trained and dedicated staff has contributed to the reduction in mortality in very and moderate preterm neonates (62). This reduction in mortality with basic measures is similar to those observed in other LMIC settings with limited resources (63–65) and in Thailand where during the same period of time, the overall neonatal mortality rate per 1,000 live births has declined from 8.41 [95%CI 5.55–10.64] to 5.61 [95%CI 3.46–7.51] (66).

However, challenges remain that need to be considered if one wants not only improve survival rates of preterm neonates but also give them a chance to reach their full potential and optimal brain development. Prioritizing the needs for more comprehensive, responsive and culturally appropriate care could occur through a consultative approach with parents, SCBU staff and doctors, but must be balanced against affordable and sustainable practices.

The intensive training and support to the staff during the first years of establishing the SCBU have enhanced the confidence of the staff in caring for preterm neonates and given hope to their parents; however, caring for very preterm neonates requires a more labor-intensive effort than that for moderate preterm neonates whilst carrying a lower chance of survival. Caring for these very preterm neonates is even more challenging in a setting where medical staff rely heavily on guidelines and “rules” as reported during the FGDs. Medical staff rely heavily on parents as well: their continuous presence is indispensable to monitor the wellbeing of the neonate, and is too often taken for granted rather than considered as a partnership in care. Although the cultural context naturally allows for a parent-infant closeness, the parents, individual strengths or emotional needs are often not considered, and the parent/health staff collaboration is limited to a task-oriented approach (e.g., mother expressing the correct amount of breastmilk at the appropriate time and feeding it to the neonate or caregiver ensuring the oxygen canula remains in place).

The control of body temperature is key for preterm neonates and should start in the delivery room (67); the Intergrowth-21st project, for example, has suggested a management protocol that spans the time of delivery to discharge from the SCBU (5). Immediate Kangaroo MOTHER CARE to improve neonatal survival has been well recognized and is fully supported by the WHO (68). Although it may seem a simple concept it is not easy to implement in a setting where only peripheral vascular access is available and there is no alternative such as umbilical- or percutaneously-inserted central catheters. Furthermore, this type of care is the mother's responsibility and requires her attentiveness while already carrying a heavy burden, as reported during the interviews. If adapted to this context, it should therefore be considered carefully and involve not only the medical team, but also the mothers and other potential caregivers.

Feeding might be perceived by mothers and medical staff alike as a stressful part of caring for preterm neonates. Complications such as vomiting feeds or abdominal distension might be worrying moments and could lead to a slower time to recover birthweight or gaining appropriate weight. This in turn may prolong SCBU stays. In addition, the timing of NGT feeds (hourly first, then spaced progressively) and the nearly exclusive use of maternal breast milk implies that mothers need to be available around the clock. Effective use of preterm feeding recommendations based on birth weight rather than gestational age can reduce early abdominal distension and vomiting (69), and may also prove beneficial to the SCBU experience and wellbeing of the mother (i.e., short term milk storage so that an alternate caregiver can provide feeds).

To improve the management of very preterm neonates with respiratory problems, the provision of 30% rather than pure oxygen to very preterm neonates might be a possibility (11). Continuous positive airway pressure (CPAP) could also improve chances of survival of preterm neonates with severe respiratory distress during the first days of life (70). It is estimated that 2 to 7% of the very preterm neonates (EGA 28–32 weeks) in this setting could have benefitted from “early CPAP” ventilation to improve the outcome of respiratory distress syndrome depending on inclusion criteria severity with the potential to avert 5–23 neonatal deaths in this prematurity category during the study period (71). However, this would require significant ongoing quality control and equipment maintenance as the prevalence of very preterm neonates eligible for CPAP is not high enough for regular practice. Additional medical staff would be needed to ensure adequate nurse to patient ratios when providing treatment.

Aside from these particulars for neonatal interventions, additional strengths of this study should be highlighted. This study is exemplary in using mixed methods that allowed for more concrete application of implementation frameworks (35, 36, 72) to direct analysis in evaluating newborn care in a resource-limited setting. Quantitative analysis used in this study approximates the effectiveness of the provision of SCBU care in this setting. Given the rapid growth of implementation research in recent years, the qualitative component of this study utilizes more codified, generalizable constructs, frameworks, and outcomes for consideration for future implementation or quality improvement studies in newborn care or—more broadly—maternal and child health programming in LMICs. This study evaluates evidence-based interventions for newborn care that has undergone a continuous improvement process and included modifications and cultural adaptations to enhance acceptability and adoption in this clinical setting.

This study adds to the literature on the broader issues related to border health. It provides instruction on health service provision for both voluntary and involuntary migrants in other global settings (33, 34), summarizes health interventions against the backdrop of a changing border context over time, and contributes data on border health in LMIC settings (15, 27, 28). Alluded to in the “Materials and methods,” facility-based care for preterm newborns was initially provided to refugees and later expanded to migrant communities (40). This tracks well with the sociodemographic transitions occurring along the Myanmar-Thailand border over this time. SMRU began its work in the 1980s in refugee camps in Thailand, providing services for persons seeking asylum from violence and civil unrest in Myanmar (41). By the time newborn facilities were established in refugee camps in 2008, this population had moved from immediate crisis to protracted displacement. Beginning in the 1990s, economic opportunities in Thailand aided in the growth of migrant communities crisscrossing the approximately 2,500 km (1,500 mile) border (73, 74). These communities provided readily accessible, unskilled labor to help fuel Thailand's local border economy and further its affluence relative to its western neighbor. This narrative contextualizes this study's findings, contrasting a refugee yet reachable population with a mobile, migrant one—both constrained by overlapping and nuanced externalities (40, 41, 52, 54, 75) that inform the adaptation, maintenance, and improvement of evidence-based provision of newborn care (12, 21, 25, 39, 42, 44, 46, 51, 76–78). Although a very specific setting, this study may provide insights to researchers, clinicians, and program managers promoting newborn health that are applicable to their context. In the least, this study shows that sustained organizational and financial commitment and continuous quality improvement can go a long way toward addressing the health needs of the vulnerable.

There are some limitations of note that pertain to the methods and the key findings. As the quantitative and qualitative analysis were performed sequentially, they represent data from different timepoints. The qualitative data are also limited as they were not conducted prospectively from the establishment of the SCBU at SMRU. Given the experience of the nurses and medics who have been working in SMRU SCBUs since their establishment, their reflections captured in FGDs lend a historical perspective to the qualitative findings. The narratives from women receiving SCBU care may be limited due to when they were collected, but they align with many of the issues related to coverage, acceptability, adoption, feasibility, and fidelity for maternal, neonatal, and infant care documented previously in this setting (23, 46, 76, 78). Given that the original mixed methods design was intended to inform best SCBU practices for this setting, the qualitative findings have been kept as a valuable contribution to the continuous improvement process for SCBU care.

Retrospective in nature, this study provides guidance in evaluating the implementation of a newborn care package but is limited in providing more in-depth instruction on systematic detail on modifications and adaptations to tailor interventions for this setting (79–81). Future studies using prospective designs can utilize theoretical and implementation frameworks to improve the quality of findings. These studies can build on the findings presented here or other quality improvement studies in LMIC settings that include and address the organizational and health worker capacity and patient needs for improved quality of newborn care (4, 27–31). As such, fidelity to the newborn care package as prescribed in the literature is not always implemented in full. Although retrospective analysis provides direction on improving care of sick newborns—alluded to above—study design and small sample sizes may limit the ability to estimate how fidelity, modification, or adaptation of specific practices affect neonatal outcomes. However, this study is strengthened by reviewing electronic medical records maintained for all neonates receiving SCBU care at SMRU and allowed for assessing the burden of disease (12, 82), care treatment patterns (83) and clinical outcomes by patients' subgroups (84). As discussed above, there may be some issues with generalizability, but this study lends some instruction and insight into border health in resource-limited settings for vulnerable populations.

## Data availability statement

The raw data supporting the conclusions of this article will be made available by the authors, without undue reservation.

## Ethics statement

The studies involving human participants were reviewed and approved by Oxford Tropical Research Ethics Committee (OXTREC 555-17) and Ethics Committee of the Faculty of Tropical Medicine, Mahidol University (TMEC 17-082). In addition, the Tak Province Community Ethics Advisory Board provided local, community approval (20171028/TCAB-14). The patients/participants provided their written informed consent to participate in this study.

## Author contributions

Concept and design: VC, AH, CT, and RM. Data acquisition: VC, AH, MD, MM, KA, PM, CC, and AP. Analysis and interpretation: VC, AH, KA, PM, and RM. Funding support: RM, FN, and PJ. Drafting: VC, AH, and RM. All authors critically reviewed and revised drafts, approve the final version submitted, and agree to be accountable to the manuscript content.
