# Supplementary material for: Born too soon in a resource-limited setting: A 10-year mixed methods review of a special care baby unit for refugees and migrants on the Myanmar-Thailand border
Source: Front Public Health. 2023 Apr 12;11:1144642. doi: 10.3389/fpubh.2023.1144642 (PMC10130587; doi:10.3389/fpubh.2023.1144642)
Supplement: Supplementary file 1 [file Table_1.DOCX]

Supplementary Material

# Supplementary Figures and Tables

Supplementary Table 1. Estimated person-day and mortality rate (per 1000) by categories of prematurity in early neonatal, late neonatal period and infancy.

|  | Person-day | Deaths | Mortality rate (per 1000) | 95% CI |
| --- | --- | --- | --- | --- |
| Overall | 209200 | 235 | 1.12 | 0.99-1.28 |
| Extreme preterm (EGA <28 weeks) | | | | |
| Early neonatal period (0-7 days) | 178 | 68 | 382.02 | 301.21-484.52 |
| Late neonatal period (8-28 days) | 95 | 7 | 73.68 | 35.13-154.56 |
| Infancy (>28 days) | 1251 | 0 | 0 | -- |
| Very preterm (EGA 28-32 weeks) | | | | |
| Early neonatal period (0-7 days) | 1091 | 62 | 56.83 | 44.31-72.89 |
| Late neonatal period (8-28 days) | 2452 | 12 | 4.89 | 2.78-8.62 |
| Infancy (>28 days) | 26077 | 5 | 0.19 | 0.08-0.46 |
| Moderate preterm (EGA 33-36 weeks) | | | | |
| Early neonatal period (0-7 days) | 8002 | 51 | 6.37 | 4.84-8.39 |
| Late neonatal period (8-28 days) | 11950 | 19 | 1.59 | 1.01-2.49 |
| Infancy (>28 days) | 1581000 | 11 | 0.07 | 0.04-0.13 |
